# The AP2/ERF Transcription Factor PgERF120 Regulates Ginsenoside Biosynthesis in Ginseng

**DOI:** 10.3390/biom14030345

**Published:** 2024-03-13

**Authors:** Yang Jiang, Qi Zhang, Zixia Zeng, Yi Wang, Mingzhu Zhao, Kangyu Wang, Meiping Zhang

**Affiliations:** 1 College of Life Science, Jilin Agricultural University, Changchun 130118, China; jiangyangyang1031@163.com (Y.J.); zhangqi0431@163.com (Q.Z.); 15179723661@163.com (Z.Z.); yi.wang@jlau.edu.cn (Y.W.); meiping.zhang@jlau.edu.cn (M.Z.); 2 Jilin Engineering Research Center Ginseng Genetic Resources Development and Utilization, Jilin Agricultural University, Changchun 130118, China

**Keywords:** *Panax ginseng*, *PgERF120* gene, ginsenoside, abiotic stress, genetic transformation

## Abstract

Ginseng (*Panax ginseng* C.A. Meyer) is a perennial herb belonging to the family *Araliaceae* and has been used for thousands of years in East Asia as an essential traditional medicine with a wide range of pharmacological activities of its main active ingredient, ginsenosides. The *AP2/ERF* gene family, widely present in plants, is a class of transcription factors capable of responding to ethylene regulation that has an influential role in regulating the synthesis of major active ingredients in medicinal plants and in response to biotic and abiotic stresses, which have not been reported in *Panax ginseng*. In this study, the *AP2/ERF* gene was localized on the ginseng chromosome, and an *AP2/ERF* gene duplication event was also discovered in *Panax ginseng*. The expression of seven *ERF* genes and three key enzyme genes related to saponin synthesis was measured by fluorescence quantitative PCR using ethylene treatment of ginseng hairy roots, and it was observed that ethylene promoted the expression of genes related to the synthesis of ginsenosides, among which the *PgERF120* gene was the most sensitive to ethylene. We analyzed the sequence features and expression patterns of the *PgERF120* gene and found that the expression of the *PgERF120* gene was specific in time and space. The *PgERF120* gene was subsequently cloned, and plant overexpression and RNA interference vectors were constructed. Ginseng adventitious roots were transformed using the *Agrobacterium tumefaciens*-mediated method to obtain transgenic ginseng hairy roots, and the gene expression, ginsenoside content and malondialdehyde content in overexpression-positive hairy roots were also analyzed. This study preliminarily verified that the *PgERF120* gene can be involved in the regulation of ginsenoside synthesis, which provides a theoretical basis for the study of functional genes in ginseng and a genetic resource for the subsequent use of synthetic biology methods to improve the yield of ginsenosides.

## 1. Introduction

*Panax ginseng* (*Panax ginseng* C.A. Meyer) is a deciduous perennial plant belonging to the *Araliaceae* family and is considered to be the king of herbal medicine [1]. The chemical components in *Panax ginseng* include saponins, polysaccharides, amino acids, peptides, etc. Ginsenosides are the distinctive bioactive components of ginseng [2], and more than 200 ginsenosides have been identified from ginseng [3,4]. Ginsenosides have the function of regulating blood pressure and restoring heart function, in addition to anti-tumor and anti-viral activities, immune regulation, cough promotion, stomach health, diuretic functions and aiding the nervous system [5,6,7,8,9]. The majority of the current supply of ginseng is sourced from conventional field cultivation, which is vulnerable to many environmental elements, such as climate, soil, pathogenic microorganisms and pests, making it challenging to guarantee quality [10]. Therefore, it is essential to identify and validate genes in ginseng that can both resist stress and regulate ginsenoside synthesis.

Plants have numerous transcription factors that are closely linked to their growth and development, metabolism and stress response [11,12,13]. The AP2/ERF gene family has been demonstrated to be one of the most important gene families in plant responses to biotic and abiotic stresses, regulating hormone signaling pathways and acting by binding to downstream cis-acting elements [14]. AP2/ERF transcription factors consist of at least one DNA-binding domain called the AP2 domain, which is a typical three-dimensional structure consisting of about 60 amino acid residues in three beta folds and one alpha helix and can be classified into five subfamilies, namely AP2, ERF, DREB, RAV and Soloist, depending on the number of structural domains and recognition sequences [15,16]. The Ethylene-Responsive Factor (ERF) subfamily plays an instrumental role in response to biotic and abiotic stresses [17] and phytohormone signaling [18].

Members of the ERF transcription factor family are also involved in the regulation of secondary metabolism biosynthesis. Lu et al. enhanced the expression of CYP71AV1, a key enzyme gene in the artemisinin biosynthesis pathway, by overexpressing the *AaERF1* and *AaERF2* genes in *Artemisia annua* and increased the accumulation of artemisinin, while the silencing of the *AaERF1* and *AaERF2* genes in *Artemisia annua* significantly inhibited the synthesis of artemisinin; thus, the *AaERF1* and *AaERF2* genes in *Artemisia annua* were positively correlated with artemisinin synthesis [19]. Menke et al. screened two AP2/ERF transcription factors (ORCA2 and ORCA3) from *Catharanthus roseus* using yeast single-heterozygosity, which were able to increase the expression of strictosidine synthase, a key enzyme gene in the periwinkle alkaloid synthesis pathway, and thus promote the synthesis of strictosidine, the precursor substance of vinblastine, which, in turn, increased the amount of vinblastine accumulation [20]. In 2019, Pan et al. found that the contents of bisindole alkaloids anhydrovinblastine and vinblastine, monoindole alkaloids ajmalicine, vindoline and catharanthine, were strongly enhanced in CrERF5-overexpressing petals, while their contents decreased in CrERF5-silenced plants. CrERF5 plays a positive role in regulating the bisindole alkaloid accumulation [21]. In 2005, De Sutter V et al. discovered that the expression levels of the AP2/ERF transcription factors NtJAP1 and NtORC1 were increased in methyl jasmonate-treated tobacco and that the expression of the promoter of the putrescine-N-methyl-transferase (PMT) synthesis gene, a key enzyme downstream of the tobacco nicotine biosynthesis pathway, was significantly upregulated, with a concomitant increase in nicotine content. As such, it is hypothesized that the AP2/ERF transcription factor NtJAP1 and NtORC1 genes positively regulate the synthesis of tobacco nicotine [22].

The AP2/ERF transcription factors are the ultimate target genes of the ethylene signaling pathway and are considered vital regulators of plant hormone signaling [23]. Real-time quantitative PCR analysis revealed that *PsAP2* gene expression was 3.7 times higher under normal conditions after 1 h of ethylene-induced treatment, thus demonstrating that this family of genes does have a hormone-responsive function [24]. Significant changes in the expression of *AP2/ERF* family members *MaERF9* and *MaERF11* were identified in banana nuts treated with ethylene. The relationship between the expression of *MaERF9* and *MaERF11* and the expression of *MaACS1* and *MaACO1*, key genes for ethylene synthesis in bananas, was also monitored, and it was found that induction with a small amount of exogenous ethylene promoted the expression of these genes, as well as the synthesis and release of ethylene, in banana nuts [25]. This is evidence of the involvement of AP2/ERF transcription factors in ethylene biosynthesis and signaling.

In this study, based on the published transcription factor data of the ginseng *AP2/ERF* gene from another laboratory, ethylene processing was performed to identify the related gene *PgERF120* gene that significantly impacts ginsenoside content, and its sequence characteristics, phylogenetic relationships and expression patterns were analyzed. The *PgERF120* gene was cloned, overexpression and RNA interference expression vectors were constructed and the *PgERF120* gene was transferred into ginseng adventitious roots using the ginseng root genetic transformation technology already established by the team. The gene expression, ginsenoside content and malondialdehyde content in the transgenic hairy roots were analyzed. This study initially revealed the role of the *PgERF120* gene in ginsenoside synthesis and in response to low-temperature stress, which can support further studies on the function of *ERF* genes in ginseng.

## 2. Materials and Methods

### 2.1. Data and Materials

The database used in this study was the ginseng unigenes database (https://www.ncbi.nlm.nih.gov/sra?linkname=bioproject_sra_all&from_uid=302556, accessed on 11 December 2015) [26] established by the Jilin Engineering Research Center Ginseng Genetic Resources Development and Utilization. The *PgERF* gene family was identified and screened by previous work [27]. The plant material used in this study was ginseng adventitious root material induced in our laboratory and cultured and propagated in B5 medium. The bacteria and vectors used in the experiment were provided by the laboratory.

### 2.2. Chromosome Localization and PgERF Gene Duplication Analysis

The laboratory identified *PgERF* genes using the ginseng unigenes database in pre-work. The *PgERF* gene was compared to the ginseng genome to determine its distribution. Using = 100% identity and a coverage length ≥ 400 bp as comparison criteria, the gene assignments of this gene family in the ginseng genome were analyzed. The location of transcripts on chromosomes was visualized using the MG2C online tool (http://www.MG2C.iask.in\/MG2C_v2.1\/index.html, accessed on 15 December 2023). The R installation package was used to analyze gene duplication events.

### 2.3. Analysis of Ethylene-Induced Ginseng Hairy Root Culture and Changes in Ginsenoside Content and Gene Expression

Ginseng root tips of 1.0 g fresh weight were cut and inoculated in 150 mL of 1/2 MS liquid medium and incubated in the dark for 24 days. The induction experiments were carried out by adding ethylene glycol at an action concentration of 50 μM as an inducer, with treatment times of 1 d, 2 d, 4 d and 6 d. Ginseng hairy roots were weighed at 0.5 g dry weight and incubated for various times to measure the monomeric saponin content by High-Performance Liquid Chromatography (HPLC). Fluorescent quantification primers were designed for the open reading frame of the fluorescent quantification gene, a SYBR Premix Ex Taq™ II (Tli RNaseH Plus) kit purchased from Bausch + Lomb Biotech (Beijing) Ltd. was used to perform the fluorescent quantification PCR reactions and the final results were calculated using the 2^−ΔΔCT^ method.

### 2.4. Characterization of the PgERF120 Gene

The basic physical and chemical properties of PgERF120 proteins were predicted with Expasy ProtParam online software (https://web.expasy.org/protparam/, accessed on 23 December 2023) and with SOPMA (https://www.genscript.com/wolf-psort.html, accessed on 23 December 2023) and SWISS-MODEL (https://swissmodel.expasy.org/, accessed on 23 December 2023), two online software programs used to analyze the secondary and tertiary structure of the *PgERF120* gene based on its amino acid sequence.

### 2.5. Expression Analysis of the PgERF120 Gene

To further analyze the expression pattern of *PgERF120* in ginseng, the expression levels of *PgERF120* in ginseng roots from four different age stages (5, 12, 18 and 25 years old), 14 different tissues of 4-year-old ginseng and 42 farm cultivars of 4-year-old ginseng roots were selected from data from previous studies in the laboratory and visualized with the use of TBtools version 1.6 [28] software.

### 2.6. Cloning of the PgERF120 Gene

Total RNA of ginseng was extracted by the TRIZOL method and reverse-transcribed into cDNA. The gene sequence of *PgERF120* was retrieved from the Jilin Ginseng Transcriptome Database, its open reading frame was identified using the NCBI ORF finder (https://www.ncbi.nlm.nih.gov/orffinder/, accessed on 25 December 2023) and primers specific for amplification were designed using Primer Premier version 5.0; the primers are as follows: F: 5′-GCTCTAGAATGGATAGAATGCACGAAAACAAC-3′, R: 5′-TCCCCCGGGTCCTAATTAGCAAGAACTTCCCAG-3′; PCR amplification was performed using cDNA as a template.

### 2.7. Construction of Recombinant Expression Vectors

The *PgERF120* gene fragment was connected with the pMD™ 18-T vector, transformed with DH5α *E. coli* receptor cells and sent to the company for sequencing; after sequencing without error, the gene fragment was connected with expression vector pBI121, which was verified by double digestion, and the overexpression vector of the target gene, PBI121-PgERF120, was successfully constructed. The interference vector was constructed by a binary vector system, and the forward- and reverse-interference fragments of the gene were obtained by amplification using specific primers, which were each ligated to the cloning vector, then inserted into the intermediate vector, pHANNIBAL. The region containing the interfering vector was cut down and inserted into vector pART27, and double-enzyme digestion was carried out to verify that the target gene interfering vector pART27-PgERF120 was successfully constructed.

### 2.8. Genetic Transformation of Recombinant Expression Vectors to Ginseng

First, we prepared genetically engineered bacteria and constructed the pBI121-PgERF120 overexpression vector and pART27-PgERF120 interference vector for *Agrobacterium tumefaciens* A4 cells. The ginseng adventitious roots were cut into 1 cm segments and placed on solid MS medium for pre-cultivation. After pre-cultivation, they were cut into smaller pieces and placed in suspension in a solution of A4 bacteria for infestation. The surface of the exosomes was aspirated, placed on solid MS medium containing acetosyringone (AS) and incubated for a total of 48 h in the dark; the exosomes were moved to 1/2 MS solid medium containing cephalosporins and incubated in dark conditions; growth was observed and recorded. When the induced hairy roots grew to 2.0 cm, they were transferred to new 1/2 MS solid medium without antibiotics for propagation until there was enough material for saponin extraction. Three-fragment PCR was used to test whether the hairy roots were positive. That is, the follow three target fragments were amplified: the target gene and the upstream vector fragment of the target gene; the target gene and the downstream vector fragment of the target gene; and the target gene.

### 2.9. Gene Expression Analysis of Overexpression-Positive Ginseng Hairy Roots

RNA was extracted from the positive tissue material and reverse-transcribed into cDNA. Fluorescent quantitative PCR was performed by using an ABI 7500 Fast Real-Time PCR System. The *GAPDH* (glyceraldehyde-3-phosphate dehydrogenase) gene was used as an internal reference gene. The gene primers were designed using Primer Premier 5.0 software for fluorescent quantitative PCR, three technical and biological replicates were set up (Table S1). The relative gene expression levels were calculated using the 2^−ΔΔCt^ formula, and the internal reference genes were normalized.

### 2.10. Ginsenoside Content of Overexpression-Positive Ginseng Hairy Roots

Total ginsenosides were determined by the vanillin-glacial acetic acid method, and content was determined by HPLC using a Waters C18 column (5 μM, 4.6 × 250 mm) at 35 °C with a 20.0 μL sample volume. Mobile phase: a. water, b. acetonitrile, gradient elution; the mobile phase flow rate was 1.0 mL/min, and the detection wavelength was 203 nm.

### 2.11. MDA Content in Ginseng Hairy Roots Treated with Hypothermia Stress

The gene sequence of *PgERF120* was matched to the publicly available Korean ginseng genome database; the *Pg_S7899.2* gene with the highest sequence homology was obtained and was found to be expressed at a 7.51-fold higher level than the control under cold stress induction. Consequently, it is hypothesized that the *PgERF120* gene is involved in cold stress response. Then, 1.0 g of wild-type ginseng hairy roots in good condition were inoculated in 1/2 MS medium and cultured for 30 days under dark conditions with constant shaking, followed by different periods of cold stress treatment at 4 °C. The ginseng hairy root samples were processed, and the absorbance was measured to calculate the malondialdehyde content.

## 3. Results

### 3.1. Chromosomal Distribution and Gene Duplication Analysis of the PgERF

**Figure 1:**
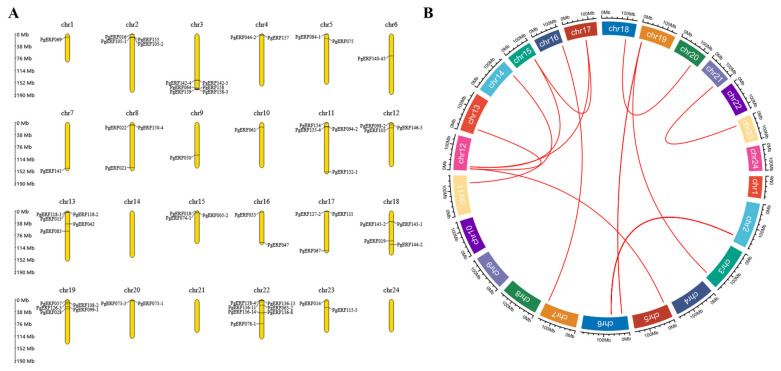
Chromosomal localization and syntenic blocks of the *AP2/ERF* gene family in *Panax ginseng*. (**A**) Chromosomal localization of the *AP2/ERF* gene family in *Panax ginseng*. (**B**) Syntenic blocks of *AP2/ERF* gene family members within the *Panax ginseng* genome. Red arcs indicate syntenic relationships between genes, Chr: chromosome, and extrachromosomal proportions represent the length of chromosomes (Mb).

After analysis, we discovered that members of the *PgERF* gene family were distributed heterogeneously across the 24 chromosomes of *Panax ginseng* (Figure 1A). The *PgERF* gene has the highest gene density on chromosome 22, with chromosomes 1, 6, 7, 9 and 10 containing only one *PgERF* gene, and no *PgERF* gene family genes were identified on chromosomes 14, 21 and 24. Gene duplication plays an influential role in the development of new functions and gene amplification, so we also analyzed the duplication events of *AP2/ERF* genes in the *Panax ginseng* genome and discovered the presence of *PgERF* gene duplication events in the *Panax ginseng* genome (Figure 1B).

### 3.2. Influence of Ethylene Treatment on the Expression of the PgERF Gene, Key Enzyme Genes and Ginsenoside Content

**Figure 2:**
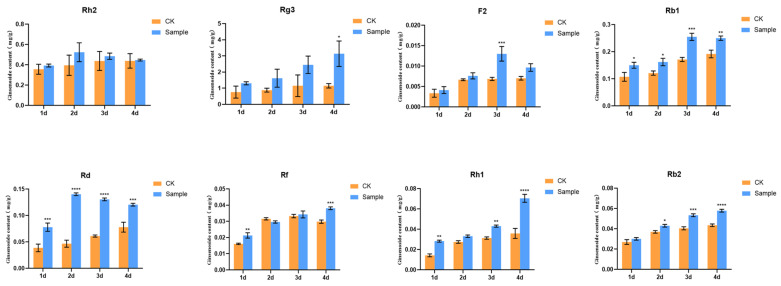
Effect of ethylene induction on eight ginsenosides content in ginseng hairy roots. The *X*-axis indicates the ethylene inducers were added to the ginseng hairy roots at four different times. The *Y*-axis indicated the ginsenoside content (mg/g). “*” indicate significant difference at *p* ≤ 0.05, “**” indicate significant difference at *p* ≤ 0.01, “***” indicate significant difference at *p* ≤ 0.001, and “****” indicate significant difference at *p* ≤ 0.0001, respectively.

Ethylene was added to the ginseng hairy roots, the ginseng material was gathered at different times of action and the content of the eight individual ginsenosides in the ethylene-induced ginseng root was measured and compared with the content of the eight individual ginsenosides in the ginseng root without ethylene treatment at the same time. The contents of the ginsenosides under ethylene treatment were significantly increased, proving that ethylene has the function of promoting the synthesis of some of the individual ginsenosides in the ginseng root cultivation system (Figure 2).

**Figure 3:**
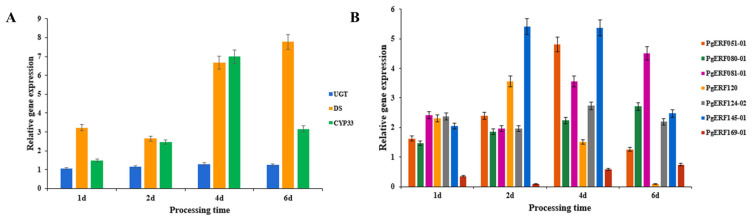
Expression changes of related genes under ethylene treatment. (**A**) Expression changes of three key enzyme genes of ginsenosides synthesis. (**B**) Expression changes of seven *PgERF* genes related to ginsenosides synthesis. Four action time nodes were set as 1 d, 2 d, 4 d, and 6 d. The action concentration of ethylene added was 50 μM.

Based on the results of a previous analysis of the correlation between the *AP2/ERF* gene family and ginsenoside content in ginseng [27], the expression levels of seven *ERF* genes and three key enzyme genes of ginsenosides biosynthesis were measured by fluorescence quantitative PCR on ethylene-treated ginseng adventitious roots. The *CYP339* gene, one of the key enzyme genes, was expressed seven times more than the control after 4 d of ethylene treatment at a concentration of 50 μM. The expression of the *DS* gene was maximum at 6 d of treatment—7.7 times that of the control. The expression of the *UGT* gene, although also upregulated at all time points, was not very significant. Among the *AP2/ERF* genes in ginseng, all genes showed upregulated expression, except for the *PgERF169-01* gene, which was downregulated (Figure 3). This resulted in a combination of changes in ginsenoside content, demonstrating that ethylene has a facilitative effect on the synthesis of ginsenosides and an effect on the expression of the *AP2/ERF* gene. The expression of the *PgERF120* gene was greatest at 2 d of treatment, then gradually decreased, with a downregulation of expression at 6 d of treatment. It was hypothesized that this gene was more sensitive to ethylene treatment or had a distinctive signaling pathway, so it was chosen for more in-depth study.

### 3.3. Sequence Analysis of the PgERF120 Gene

**Figure 4:**
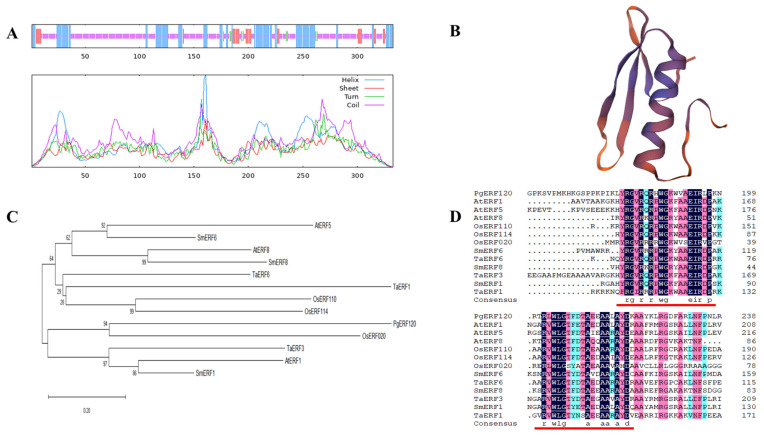
Characterization of the *PgERF120* gene. (**A**) Secondary structure analysis of the ginseng PgERF120 protein. Blue, green, purple and red lines represent α-helix, β-turn, random coil and extended chain. (**B**) Tertiary structure of PgERF120 protein. (**C**) Evolutionary relationships between *PgERF120* and some *ERF* gene family members of the remaining species. (**D**) Comparison of amino acid sequences of the *PgERF* with protein sequences of other species. The red line marks the ERF structural domain.

The *PgERF120* gene is 1955 bp in length, encoding 333 amino acids, with a molecular weight of 35.68 kDa and an isoelectric point (pI) of 7.02. The secondary structure of the PgERF120 protein contains 92 alpha helices (27.63%), 9 beta helices (2.70%), 205 random coils (61.56%) and 27 extended shares (8.11%) (Figure 4A). Three-level protein structure prediction revealed that PgERF120 has the typical three-dimensional structure of AP2/ERF family proteins (Figure 4B). To reveal the evolutionary relationships between *ERF* genes in different species, protein sequences of ERF family members from *Oryza sativa* (Os), *Arabidopsis thaliana* (At), *Salvia miltiorrhiza* (Sm) and *Triticum aestivum* L. (Ta) were downloaded. A phylogenetic tree was constructed using the PgERF120 protein sequence, and 12 ERF protein sequences from the remaining four species (Figure 4C) and PgERF120 and OsERF020 were most closely related evolutionarily. At the protein level, PgERF120 has a high degree of similarity to the amino acid sequences of the remaining four plants involved in the comparison (Figure 4D).

### 3.4. Analysis of the Expression Pattern of PgERF120 in Ginseng

**Figure 5:**
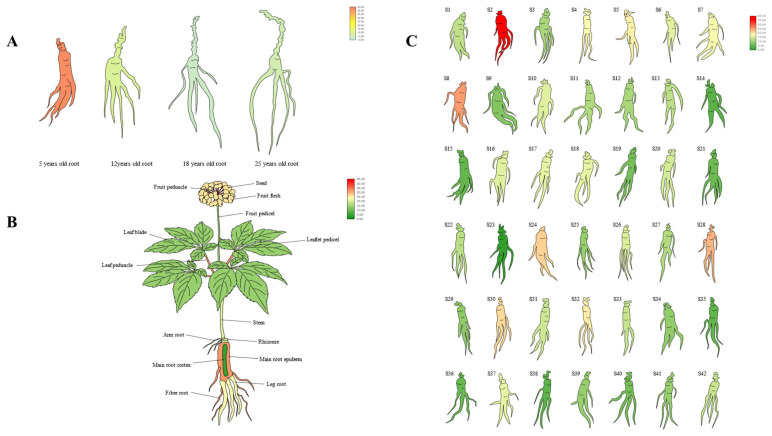
Heatmaps analysis spatiotemporal expression patterns of the *PgERF120* gene in ginseng. (**A**) The *PgERF120* gene expressed in the 4 different aged stages (5, 12, 18, 25 years-old) of ginseng roots. (**B**) The *PgERF120* gene expressed in the 14 different tissues of 4-year-old ginseng. (**C**) The *PgERF120* gene expressed in the 42 farm cultivars of 4-year-old ginseng roots.

To identify the expression pattern of *PgERF120* in ginseng, we extracted *PgERF120* gene expression data from four ginseng roots of different age stages, 14 different tissues of 4-year-old ginseng and 42 farm cultivars of 4-year-old ginseng and mapped the gene expression in a heatmap. Among ginseng roots of four different age stages, *PgERF120* gene expression levels were at the highest in 5-year-old ginseng roots and at the lowest in 18-year-old ginseng roots (Figure 5A). In 14 different tissues of 4-year-old ginseng, the *PgERF120* gene was expressed in all tissues, with high expression in the fruit peduncle and the least expression in seeds (Figure 5B). In 42 farm cultivars of 4-year-old ginseng roots, the *PgERF120* gene was highly expressed in S2 and S8 and less so in S23 (Figure 5C).

### 3.5. Cloning and Vector Construction of the PgERF120 Gene

**Figure 6:**
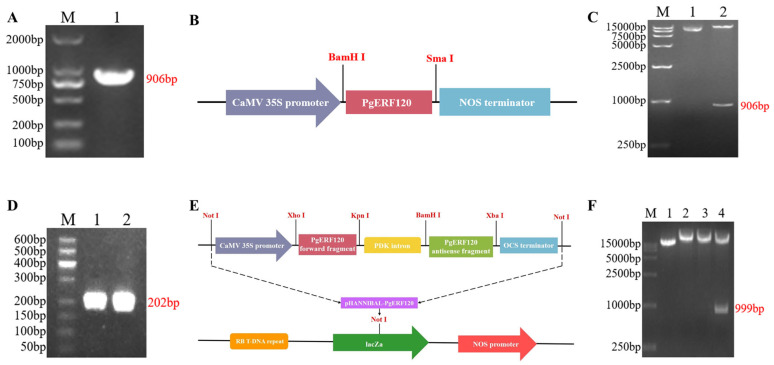
Cloning and vector construction of *PgERF120* gene. (**A**) PCR amplification electropherogram of *PgERF120*. M: DL2000 Marker; 1: PCR amplification result. (**B**) Construction of expression vector pBI121-PgERF120. (**C**) Enzyme digestion verification of pBI121-PgERF120 recombinant plasmid. M: DL15000 Marker; 1: recombinant plasmid; 2: recombinant plasmid *BamH*I and *Sma*I double digestion. (**D**) PCR amplification of *PgERF120* interfering fragment. M: DL500 Marker; 1: forward interfering fragment; 2: reverse interfering fragment. (**E**) Construction of expression vector pHANNIBAL-PgERF12. (**F**) Enzymatic validation of the pHANNIBAL-PgERF120 recombinant plasmid. M: DL15000 Marker; 1: recombinant plasmid; 2, 3: recombinant plasmid interference fragment enzymatic cleavage. 4: recombinant plasmid *Spe*I and *Hind*III double digestion.

With cDNA as a template, the *PgERF120* gene was successfully cloned, and the fragment length was 906 bp (Figure 6A). The recombinant plasmid pBI121-PgERF120 was double-digested with *BamH*I and *Sma*I, and the length of the cut bands matched with the target fragment, so the overexpression vector was successfully constructed (Figure 6B,C). The forward- and reverse-interfering fragments of the target gene were successfully obtained with a length of 202 bp (Figure 6D). At the same time, it was verified by double-enzyme digestion that the cut fragment matched the actual fragment, and the interference vector of the target gene was constructed successfully (Figure 6E,F).

### 3.6. Genetic Transformation of Ginseng with Recombinant Plasmids

**Figure 7:**
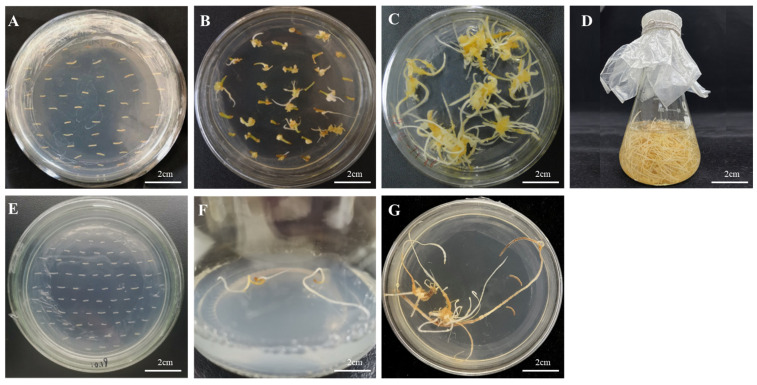
Induction of hairy roots by *PgERF120* gene overexpression vector and interference vector transformation. (**A**) Co-culture of overexpression vector transformed adventitious roots. (**B**) Induction of overexpression vector transformed hairy roots. (**C**) Culture of overexpression vector transformed hairy roots. (**D**) Expansion of overexpression vector transformed hairy roots. (**E**) Interference vector transformed adventitious roots. (**F**) Interference vector transformed hairy roots induction. (**G**) Interference vector transformed hairy roots culture.

**Figure 8:**
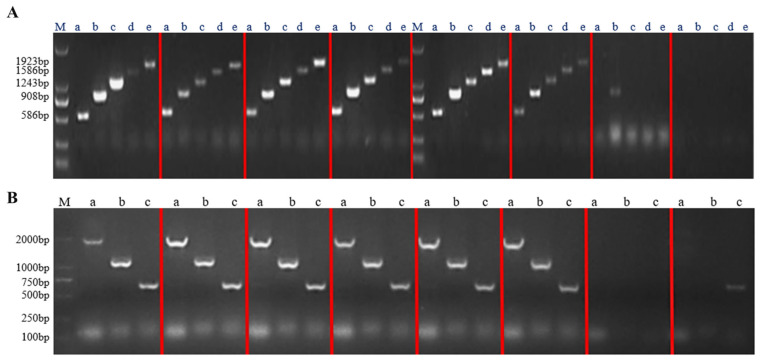
The *PgERF120* gene overexpression and interference plant assays. (**A**) Detection of overexpression of hairy root positive plants. M: DL2000 Maker, a–e: hairy root PCR assay. (**B**) Detection of RNA interference in hairy root positive plants. M: DL2000 Maker, a–c: hairy root PCR assay.

The recombinant plasmids of the successfully constructed overexpression vector and RNA interference vector were transfected into *Agrobacterium tumefaciens* A4 cells. After infesting the adventitious roots of *Panax ginseng* with A4, the roots were cultivated in the dark for four weeks. The hairy roots were cut when they reached 1–2 cm in length and transferred to a new solid 1/2 MS medium for succession (Figure 7). A total of 42 overexpression hairy root asexual lines and 23 RNA interference hairy root asexual lines were obtained. The genomic DNA of the hairy roots was excised, and a three-stage PCR assay was conducted using wild-type hairy roots as a negative control and water as a blank control (Figure 8); the results proved that a successful transgenic ginseng hairy root asexual line was obtained.

### 3.7. Determination of Gene Expression in Overexpression-Positive Ginseng Hairy Roots

**Figure 9:**
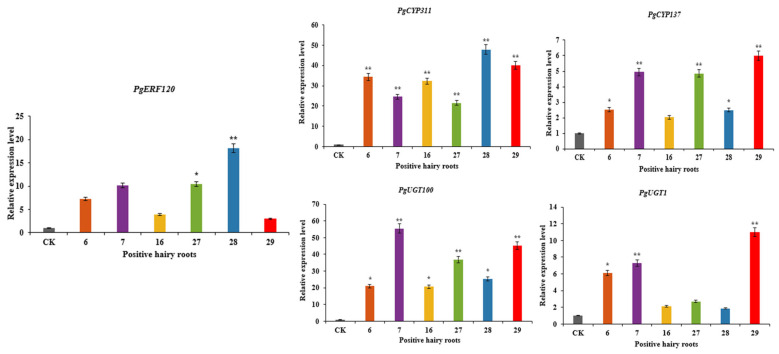
Relative expression level of genes in overexpression-positive ginseng hairy roots. The *X*-axis indicates the overexpression-positive ginseng hairy roots lineage. The *Y*-axis indicated the genes relative expression levels. “*” indicate significant difference at *p* ≤ 0.05, “**” indicate significant difference at *p* ≤ 0.01, respectively.

Positive hairy root asexual lines overexpressing *PgERF120* were labelled 6, 7, 16, 27, 28 and 29, and the relative expression levels of *PgERF120* in overexpression-positive hairy roots were measured using wild-type hairy roots as a control (Figure 9). The relative expression levels of the *PgERF120* gene were all increased in the positive hairy root asexual lines compared to the wild-type hairy roots, with the smallest change in expression in hairy root asexual line 29, which was 2.99 times that of the control, and the most significant change in expression in hairy root asexual line 28, which was 18.16 times that of the control. This indicates that hairy root asexual lines overexpressing the *PgERF120* gene were successfully obtained.

The expression levels of *PgCYP137*, *PgCYP311*, *PgUGT100* and *PgUGT1* genes were differentially upregulated in overexpression-positive ginseng hairy roots, with the most significant increase in *PgCYP311* expression, which was extremely significantly higher in all hairy root asexual lines assayed relative to the control (Figure 9), suggesting that *PgCYP137*, *PgCYP311*, *PgUGT100* and *PgUGT1* genes may be involved in the PgERF120-mediated saponin synthesis pathway.

### 3.8. Determination of Ginsenoside Content in Overexpression-Positive Ginseng Hairy Roots

**Figure 10:**
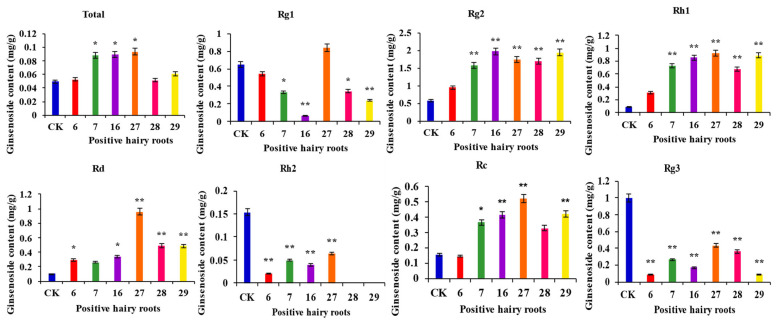
Overexpression the *PgERF120* gene of ginsenosides content of ginseng hairy root. The *X*-axis indicates the overexpression-positive ginseng hairy roots lineage. The *Y*-axis indicated the ginsenosides content (mg/g). “*” indicate significant difference at *p* ≤ 0.05, “**” indicate significant difference at *p* ≤ 0.01, respectively.

Determination of the ginsenoside content of PgERF120-positive hairy root asexual lines and wild-type hairy roots (Figure 10). The content of the four monosaponins Rc, Rd, Rh1 and Rg2 was significantly increased, and the content of monosaponins Rg1, Rh2 and Rg3 was significantly reduced in the transgenic hairy roots overexpressing the *PgERF120* gene. The total ginsenoside content was significantly higher in the three transgenic hairy root asexual lines (Nos. 7, 16 and 27) overexpressing the *PgERF120* gene than in the control. These results indicated that the *PgERF120* gene could regulate the biosynthesis of ginsenosides.

### 3.9. Determination of MDA Content in Ginseng Hairy Roots Treated with Cold Stress

**Figure 11:**
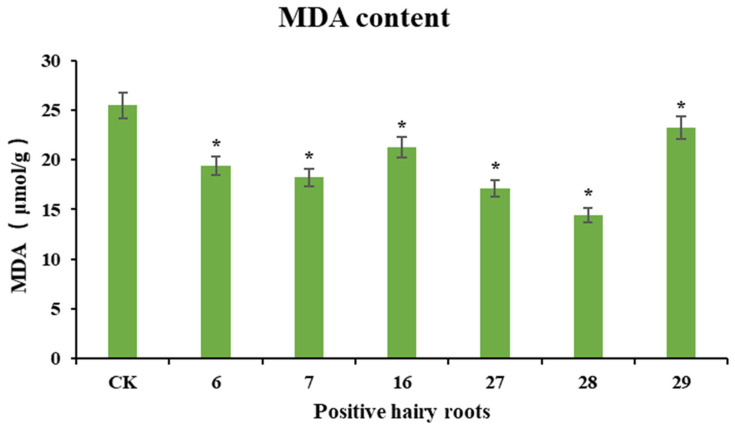
MDA content in cold-stressed ginseng hairy roots. The *X*-axis indicates the overexpression-positive ginseng hairy roots lineage. The *Y*-axis indicated the MDA content (μmol/g). “*” indicate significant difference at *p* ≤ 0.05, respectively.

Cold stress causes peroxidative damage to cell membranes, and the level of malondialdehyde is often used as an indicator of cell membrane permeability and membrane lipid peroxidation. To investigate the resistance of PgERF120-positive ginseng hairy roots to cold stress, wild-type hairy roots were used as a control, overexpressing positive hairy roots were subjected to cold stress treatment and changes in malondialdehyde content in positive hairy roots after the stress treatment were measured (Figure 11). After the wild-type hairy roots and overexpression-positive hairy roots were jointly treated with cold stress at 4 °C for 12 h, the malondialdehyde content detected in the overexpression-positive hairy roots was significantly lower than that in the control group, consistent with the trend of *PgERF120* gene expression in different hairy root asexual lines, suggesting that the overexpression of *PgERF120* improved the resistance of ginseng hairy roots to cold stress.

## 4. Discussion

The AP2/ERF transcription factor family has been extensively researched in a variety of plants, such as *Arabidopsis* [29], *Fagopyum tataricum* [30], *Hordeum vulgare* [31] and *Rhododendron* [32]. We targeted the *PgERF* gene to chromosome 24 pairs in *Panax ginseng*, and 11 duplication events were identified in the 24 pairs of ginseng chromosomes (Figure 1).

As an endogenous plant hormone, ethylene has the function of regulating plant growth and development and secondary metabolism [33]. The AP2/ERF transcription factor has a function in response to ethylene signaling [34]. We used exogenous ethylene to treat ginseng hairy roots, detected changes in the content of ginsenosides and found that the content of ginsenosides in the treated group increased significantly compared to the control group, demonstrating that ethylene can promote the synthesis of ginsenosides. The results of fluorescence quantification showed that the expression of some *AP2/ERF* genes in ginseng hairy roots was upregulated by ethylene treatment, demonstrating that ethylene has the function of regulating the expression of part of *AP2/ERF* genes. Exogenous ethylene treatment of ginseng hairy roots significantly increased the content of monomeric saponins and the expression of *AP2/ERF* genes, indicating that *AP2/ERF* genes can promote the biosynthesis of ginsenosides, a result consistent with the ability of *AP2/ERF* genes to regulate the synthesis of active ingredients in medicinal plants [35,36,37,38].

Dammarendiol synthase (DDS) has been shown to cyclize 2,3-oxo-squalene to produce dammarendiol [39]. In 2011, Han [40] co-expressed *PgDDS* and *PgCYP716A47* in yeast and found that protopanaxadiol-type sapogenins (PPDs) could be produced without the addition of dammarendiol and verified that *CYP716A47* could catalyze the production of PPD from dammarendiol by in vitro enzymatic activity assays. In 2019, Gwak [41] co-transformed *PgDDS*, *PgCYP716A47* and *PgCYP716A53v2* (*PgCYP137*) into tobacco and produced protopanaxatriol-type sapogenins (PPT) in transgenic tobacco, indicating that *CYP716A53v2* can catalyze PPD to produce PPT. In 2014, Yan [42] heterologously expressed the *UGTPg1* (*PgUGT1*) gene in yeast and found that the UGTPg1-encoded protein could catalyze the production of rare ginsenoside CK from PPD as a substrate, as well as another novel compound, 20S-O-β-(D-glucosyl)-dammarenediol II (DMG). *UGTPg1* catalyzed the production of DMG from dammarenediol, which, in turn, was catalyzed by *CYP716A47* to produce CK. When PPD and dammarenediol were present together, dammarenediol was preferentially selected as the substrate to exert catalytic activity; this study also verified the catalytic activity of *UGTPg1* in converting Rg3 to Rd. In 2015, Wei [43] demonstrated that the protein encoding the *UGTPg100* (*PgUGT100*) gene catalyzes the production of Rh1 from PPT by specifically glycosylating the C6-OH of PPT.

**Figure 12:**
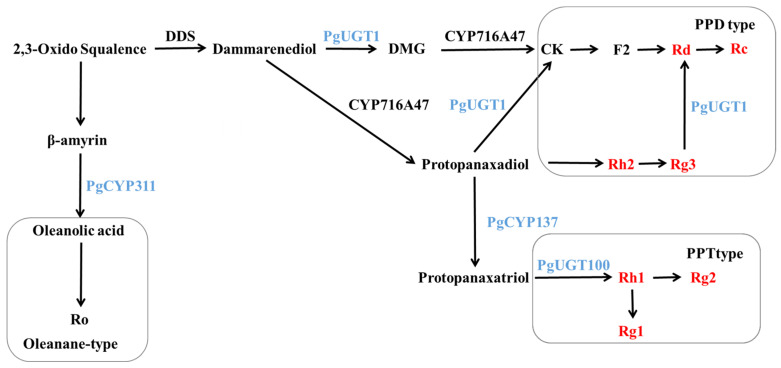
Biosynthetic pathway of ginsenosides.

The above previous research results are shown in Figure 12. The results of the study showed that the contents of Rh2 and Rg3 in positive ginseng hairy roots overexpressing the *PgERF120* gene were significantly reduced, while the contents of Rd and Rc were greatly increased. It is therefore speculated that the overexpression of the *PgERF120* gene may have upregulated the expression of the *PgUGT1* gene, resulting in a greater conversion of Rh2 and Rg3 to Rd, then to Rc, leading to a reduction in the contents of Rh2 and Rg3. It has been shown that diol-type saponins compete with triol-type glycoside PPT [44], from which it can also be inferred that PPD is converted more into PPT due to upregulation of *PgCYP137* gene expression, resulting in less substrate for the synthesis of Rh2 and Rg3, which leads to a decrease in Rh2 and Rg3 contents. In contrast, the increased amounts of Rd and Rc may be due to the progressive production of CK, F2, Rd and Rc from the DMG pathway catalyzed by the protein encoded by *PgUGT1*, which increases the amounts of Rd and Rc. The increase in the contents of Rh1 and Rg2 was due to the fact that the content of PPT, a precursor for the synthesis of triol-type saponins, may have increased with the expression of *PgCYP137*, but no remarkable changes were detected in its content as an intermediate product that was continuously consumed to produce the next level of product. Rh1 is located at the Rg1 and Rg2 typing sites, and the reduced level of Rg1 is presumed to be the result of overexpression of the *PgERF120* gene, causing a key enzyme of unknown function to catalyze more conversion of Rh1 to Rg2 or to inhibit the conversion of Rh1 to Rg1. The present study also detected that *PgCYP311* (*PgCYP716A52v2*) was upregulated by *PgERF120* and that the protein encoded by *CYP716A52v2* could catalyze the oxidation of β-coumarin to produce Ro [45]. The present experiment did not measure the Ro content, and it was not possible to verify whether the changes in Ro content in *PgERF120* ginseng hairy roots were consistent with previous studies. We will fill this gap in our subsequent work. Overall, the *PgERF120* gene can regulate the biosynthesis of ginsenosides and is of practical value for ginsenoside production, but its transcriptional regulatory mechanism is not yet clear and needs to be proven in further studies.

An increasing number of studies have shown that AP2/ERF transcription factors play an important role in plant cold stress response. Malondialdehyde, a product of membrane lipid peroxidation, can indicate the degree of plant damage and is often used as a physiological indicator of plant resistance to cold [46]. Overexpression of *MbERF11* in Arabidopsis increased the cold resistance of transgenic Arabidopsis and also significantly reduced malondialdehyde content [47]; this finding is consistent with our findings. The levels of malondialdehyde in all six tested transgenic hairy root asexual lines overexpressing *PgERF120* were significantly lower than the control; presumably, *PgERF120* enhances cold tolerance by reducing the extent of oxidative damage to plant cells under low-temperature stress.

The *PgERF120* gene both regulates ginsenoside biosynthesis and participates in cold stress response to improve the resistance of ginseng hairy roots to cold stress, reflecting the diversity of gene functions. We have not yet determined the ginsenoside contents and expression of key enzyme genes in RNA interference-positive hairy roots due to the constraints of the culture cycle of positive material. We will refine these data in subsequent studies and look forward to clarifying the function of the *PgERF120* gene in ginsenoside biosynthesis.

## 5. Conclusions

In this study, we found ginseng hairy roots treated with ethylene showed a significant increase in the content of partial monomeric saponins and that the *AP2/ERF* gene in ginseng could respond to ethylene signals and correlates remarkably with changes in ginsenoside content, indicating that the *AP2/ERF* gene could promote ginsenoside synthesis. We successfully cloned the *PgERF120* gene, constructed the plant overexpression vector and the RNA interference expression vector and transformed ginseng adventitious roots. Nine overexpression-positive hairy root asexual lines and six RNA interference-positive hairy root asexual lines were obtained, and the overexpression-positive hairy root saponin content and related gene expression levels were detected, indicating that *PgERF120* can regulate ginsenoside biosynthesis by affecting the expression of key enzyme genes. We identified and cloned an *ERF* gene (*PgERF120*), which is closely related to ginsenoside biosynthesis, verified the function of the *PgERF120* gene and preliminarily analyzed the molecular mechanism of the *ERF* gene in regulating ginsenoside biosynthesis. This lays a foundation for the study of the regulatory network of ginsenoside synthesis by *ERF* gene family members, provides a theoretical basis for the further improvement of the ginsenoside synthesis pathway and provides genetic resources and a theoretical basis for the realization of factory production of ginsenoside in plant cells.

## Data Availability

All data generated or analyzed during this study are included in this published article. All plant materials are available through corresponding authors upon request.

## Supplementary Materials

The following supporting information can be downloaded at: https://www.mdpi.com/article/10.3390/biom14030345/s1, Table S1: Primers used in the experiments.
